# Vaccine-Preventable Outbreak of Acute Respiratory Illness and Pneumonia Associated with Adenovirus at a U.S. Marine Corps Training Center

**Published:** 2025-07-20

**Authors:** Lynn A. Van Airsdale, Jacqueline M. Peretti, Asha J. Riegodedios, Aliye Z. Sanou, Lisa A. Pearse

**Affiliations:** Navy Environmental and Preventive Medicine Unit (NEPMU) FIVE, San Diego, CA: LCDR Van Airsdale, LCDR Peretti; Navy Marine Corps Force Health Protection Command (NMCFHPC), Portsmouth, VA: CDR Sanou, Ms. Riegodedios, Dr. Pearse

## Abstract

Adenovirus outbreaks have long been a cause of acute respiratory disease, hospitalization, and death in otherwise young, healthy military recruits. The administration of oral, live attenuated adenovirus (AdV) vaccine against AdV types 4 and 7 has been critical in preventing outbreaks in this population. ^1 - 4^ In early July 2024, a spike in recruit hospitalizations for AdV pneumonia was recognized at the Marine Corps Recruit Depot (MCRD) San Diego, and an outbreak investigation commenced. From July 1 through September 23, 2024, a total of 212 AdV cases, including 28 hospitalizations, were identified among trainees and staff. Non-pharmaceutical interventions, including aggressive environmental cleaning, separation of sick and well recruits, and masking, were implemented. The outbreak was not appreciably slowed, however, until AdV vaccine administration was advanced from day 11 to day 1 post-arrival of recruits to MCRD San Diego. This outbreak report demonstrates that early AdV vaccination for newly arriving recruits is an effective and essential step in preventing AdV morbidity and mortality in a recruit training setting.

Military recruit populations are uniquely susceptible to acute respiratory disease (ARD) outbreaks due to the rapid introduction of large numbers of people from a broad geographic catchment area into crowded, congregate, and high stress living conditions. ^3^ Historical studies of ARD show that up to 80% of febrile ARD cases in recruits are due to adenovirus (AdV), with 20% resulting in hospitalization. ^5^ Serotypes 4 and 7 were most common, repeatedly resulting in military recruit outbreaks. ^6^

As a result of high rates of morbidity and disruption of recruit training, the U.S. military developed and implemented a live, oral vaccine against AdV serotypes 4 and 7 starting in the 1970s, through the 1990s, that successfully reduced respiratory illnesses. ^7^ Febrile respiratory illness in vaccinated recruit training sites decreased by 50% and AdV infection decreased by more than 90%. ^3 , 4 , 8 , 9^

Vaccine production was halted by the sole manufacturer in 1995 and total depletion of AdV vaccine supply occurred in 1999. Between 1999 and 2011, multiple large outbreaks of AdV resurfaced in recruit training centers across the U.S., ^10 - 13^ resulting in 8 deaths associated with AdV infection. ^1^ AdV vaccine was reintroduced to the recruit population in 2011, proving to be 99.3% effective, and within 2 years there was a 100-fold decline in AdV disease burden. ^14^ Sporadic outbreaks have subsequently occurred, primarily affecting populations where adenovirus vaccine is not routinely administered. ^15 - 17^

At Marine Corps Recruit Depot (MCRD) San Diego, new recruits arrive weekly and are placed into platoons that comprise companies. Companies then train together for a 12-week training cycle. MCRD's staggered training cycle maximizes training efficiency, and involves ongoing close contact, high density living environments, and potential exposure to, and spread of, infectious diseases. Prior to training commencement, recruits proceed through “receiving week,” which begins day 1 post-arrival and includes a medical portion for laboratory testing of vaccine titers, G6PD (glucose-6-phosphate dehydrogenase) status, blood typing, gonorrhea and chlamydia testing, HIV and hepatitis screening, and universal pregnancy testing for female recruits. To avoid exposing pregnant and separating recruits to live vaccines, vaccines were historically administered day 11 post-arrival, after all laboratory results were received.

**What are the new findings?**

Despite the availability and widespread use of effective vaccines during recruit training, adenovirus outbreaks remain a significant threat to military recruits if the vaccine is not administered expediently, upon arrival to the recruit training center.

**What is the impact on readiness and force health protection?**

Adenovirus outbreaks can occur in military recruit environments when vaccination is not accomplished promptly after arrival. Recruit vaccination prior to, or very soon after, arrival to a military recruit setting minimizes the impacts of adenovirus by preventing disease outbreaks, medical separations, and training disruption.

In early July 2024, local military public health assets were alerted to a spike in hospitalized AdV pneumonia cases of MCRD San Diego recruits. This triggered an outbreak investigation to identify the reason for the increased number of cases and to implement mitigation measures. This report describes the investigation and findings of a major outbreak of AdV since the re-introduction of the AdV vaccine in 2011.

## Methods

In early July 2024, MCRD San Diego experienced 9 hospitalized AdV pneumonia cases within a 2-week period, accompanied by a notable increase in outpatient ARD cases. An outbreak investigation was initiated on July 16. A case was defined as an outpatient or inpatient MCRD San Diego recruit or training site staff member with AdV detected on multiplex respiratory pathogen PCR (polymerase chain reaction; BIOFIRE Respiratory 2.1) on or after July 1, 2024.

A line listing of cases was maintained in Microsoft Excel and managed in Microsoft Teams (with access controlled) to promote transparency within the outbreak response team. The Military Health System Electronic Health Record–Generation Next (MHS GENESIS) and Naval Medical Center San Diego (NMCSD) Nurse of the Day report were used to populate the line list data, including demographics (e.g., recruit or staff, age, sex, training date, company), AdV vaccination date, date of symptom onset, date of first clinical visit, hospitalization status including date of admission and discharge, pneumonia diagnosis, AdV laboratory result, and other co-infections. Other co-infections were determined on the same multiplex respiratory pathogen PCR that detected AdV.

Due to delays in seeking care, particularly at the beginning of the outbreak, epidemiological curves were created based on date of symptom onset as well as date of initial clinic visit. Although the case definition represents those testing positive on or after July 1, the symptom onset of cases with positive laboratory tests dated back to as early as June 10, which is when this outbreak surveillance period began.

Illness severity was monitored based on hospitalization status, number of days hospitalized, and need for repeated hospitalization. Attack rates by company were calculated using company population estimates from the beginning of each training cohort.

Vaccination and symptom onset association was calculated using the vaccine administration date and symptom onset date. Vaccine protection analysis compared the rates of AdV between non-immune—defined as unvaccinated or within 14 days post-vaccination—and immune individuals—defined as symptom onset more than 14 days post-vaccination. This was calculated based on crude attack rates among selected recruit companies (Charlie, Fox, Lima, Bravo, Echo, India, Delta) who had similar chances of exposure (e.g., started training after the outbreak began and before acceleration of the vaccine schedule), representing a total of 4,500 recruits.

The end of the outbreak was defined as 28 days, or 2 maximum incubation periods, after the last symptomatic patient that resulted in an inpatient admission, and outpatient AdV case counts that remained below baseline. Baseline outpatient AdV case counts were determined through evaluation of historic records from the Discern Reporting Portal in MHS GENESIS to be 2 cases per 7-day timeframe.

## Results

**FIGURE 1.**
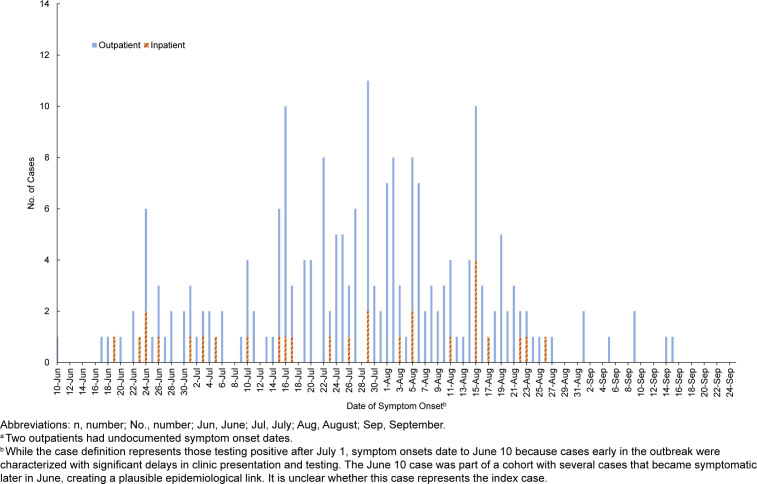
Distribution of Adenovirus Cases by Symptom Onset Date and Patient Status (n=210) ^a^

The epidemiological curve, based on symptom onset, demonstrated a propagated source outbreak that occurred from June 10 to September 15 **(Figure 1)** . A total of 212 AdV cases from MCRD San Diego were identified. Twenty-eight of the MCRD San Diego AdV cases required hospital admission, and 3 required ICU admission. There were no fatalities. Recruits accounted for 96.7% of the AdV outbreak cases, with the remainder in staff members ( **Table 1** ). A majority of the AdV cases also tested positive for other infectious etiologies, such as rhinovirus / enterovirus, seasonal coronaviruses, COVID-19, parainfluenza, H. metapneumovirus, influenza A and B, group A *Streptococcus* , and *M. pneumoniae* .

**TABLE 1.** Distribution of Adenovirus Cases by Selected Factors

Factor	Cases	Total
	No.	%
Status		
Recruit	205	96.7
Staff	7	3.3
Sex		
Female	18	8.5
Male	194	91.5
Severity indicators		
Pneumonia	79	37.3
Inpatients	28	13.2
ICU (out of all inpatients)	3	10.7
Re-admitted (out of all inpatients)	6	21.4
Number of days in hospital (average)	8.6 days	
Hospitalization rate	13.2 (%)	
Additional laboratory-specific etiologies		
Rhinovirus / enterovirus detected	123	58
COVID-19 / SARS-CoV-2 detected	38	17.9
Other etiologies detected	158	74.5
Average time from vaccination date to symptom onset date ^a^		
All cases	0.9 days (SD 9.4 days)	
Outpatients	1.1 days (SD 8.5 days)	
Inpatients	-0.4 days (SD 6.4 days)	

Abbreviations: No., number; ICU, intensive care unit; COVID-19, coronavirus disease 2019; SARS-CoV-2, severe acute respiratory syndrome coronavirus 2; SD, standard deviation.

a Calculation includes only recruits in training; recruit center staff and recruits dropping to ‘holding’ company not included.

**FIGURE 2.**
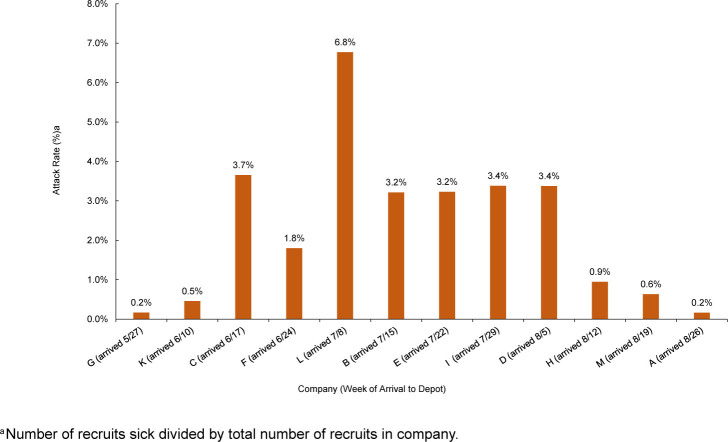
Attack Rates ^a^ of Adenovirus Illness During the Adenovirus Outbreak at Marine Corps Recruit Depot San Diego, by Company, June–September 2024

Attack rates, by company and date of arrival to recruit training, are shown in **Figure 2** . The outbreak affected 7 companies, with an average rate of illness of 3.6% per company. The company with the highest rate (6.8%) of illness arrived at MCRD San Diego during the week of July 8. Average length of time from AdV vaccination to date of symptom onset was 1 day **(Table 1)** .

The AdV vaccination schedule was accelerated on August 14. The first company to receive the vaccination day 1 post-arrival exhibited an attack rate of 0.95%, a 4-fold decrease compared to the average of the previous 7 companies. Ultimately, the overall attack rate of AdV among non-immune individuals was 3.3%, compared with 0.1% for those who were considered immune, representing a 35.6-fold difference **(Table 2)** . While a few recruits developed AdV 14 days after vaccination, none required admission.

**TABLE 2.** Risk of Illness Among Non-Immune and Immune Recruits Prior to Advanced Vaccine Schedule

Case and Immunity ^a^ Status	Cases	Attack Rate ^b^	Risk Ratio
	No.	%	
All cases			
Non-immune	147	3.3	35.6
Immune	4	0.1	
Outpatients only			
Non-immune	128	2.8	31.0
Immune	4	0.1	
Inpatients only			
Non-immune	19	0.4	—
Immune	0	0	

Abbreviations: No., number; n, number.

a Non-immune indicates symptom onset before vaccination through day 14 post-vaccination. Immune indicates symptom onset after 14 days post-vaccination.

b The estimated susceptible recruit population (n=4,500) was used to determine the attack rate among cases with non-immunity; non-immune cases were excluded from the susceptible population (n=4,353) to estimate attack rates for cases with immunity.

The average length of hospital stay (including re-admission time) was 8.6 days. As of February 28, 2025, 18 hospitalized recruits were returned to active duty as fit for full duty, and 10 recruits were separated for health reasons. The last AdV inpatient admission occurred on August 26, 2024. The end of the outbreak was declared on September 23, 2024, based on the criteria of 28 days after the last symptomatic patient with inpatient admission and out-patient adenovirus case counts remaining below baseline.

## Discussion

Adenovirus, a vaccine-preventable disease, has historically led to significant morbidity, mortality, and training disruptions in U.S. military training sites. ^5 - 7 , 18^ MCRD San Diego experienced an introduction of AdV in June 2024 that opportunistically spread through an under-vaccinated population of recruits during the 26-day period from recruit arrival to full AdV protection, defined as 14 days post-vaccination. The virus spread readily between the training companies, introduced into new companies as they arrived, until vaccine administration was advanced to day 1 post-arrival at the training center.

Upon discovery of the AdV outbreak, preventive medicine and public health entities rapidly engaged with the MCRD clinic, as well as recruit training staff, drill instructors, and MCRD dining hall staff, to communicate disease education and environmental risk recommendations. Non-pharmaceutical interventions—including enhanced hygiene and disinfection protocols, increased emphasis on hand hygiene, segregation of ill recruits, improved berthing air circulation, and food service modifications to halt self-service—were quickly introduced. Weekly habitability inspections were conducted by public health personnel, to reinforce the recommendations. Despite these interventions, the outbreak continued to spread.

Early in the outbreak it was noted that AdV vaccination was being administered day 11 post-arrival, to allow for pregnancy testing of accessioning females, in addition to assessment of vaccine titers. A joint Department of Defense (DOD) regulation, *Immunizations and Chemoprophylaxis for the Prevention of Infectious Diseases* , ^19^ prescribes immunizations for prevention of infectious diseases and provides general principles, procedures, policies, and responsibilities but does not dictate precise vaccination schedules. Implementation of the regulation varies among military training sites, with most training sites administering AdV vaccine by day 6 post-arrival.

After reviewing other training sites' vaccine timing schedules and determining time required for complete immunity, the preventive medicine and public health entities involved in this outbreak response recommended shifting AdV vaccine administration, along with other standard vaccines, from day 11 to day 1 post-arrival. This became a top priority for outbreak control. On August 14, the AdV vaccination schedule was advanced to day 1 post-arrival.

While the concern of vaccinating women with a live virus vaccine is legitimate, pregnancy testing is not required by instruction in DOD nor U.S. Navy policy. ^19^ Although, to date, there have been no documented adverse pregnancy outcomes due to AdV vaccination, there is a theoretical risk to the fetus with live vaccine administration, thus live vaccines are a general contraindication during pregnancy. ^20 - 22^

Two weeks after initiation of the expedited vaccine schedule, overall incidence was rapidly declining. At that time, it was found that the majority of new infections were in recruits who had missed the initial AdV vaccination day and received no AdV vaccine. Once this was discovered, the medical team at MCRD identified those recruits, who had been removed from training but remained on base for medical reasons or for administrative separation, and ensured vaccination completion in this population.

Interestingly, nearly 75% of cases had co-infections with other respiratory pathogens, most notably seasonal coronaviruses, COVID-19, and rhinovirus / enterovirus. This finding suggests that infection with AdV may increase susceptibility to other viruses, although more research is needed to better interpret this finding, which has not been identified in previous AdV outbreaks. Newly increased testing sensitivity associated with multiplex respiratory pathogen PCR availability may have been a factor in co-infection identification during this outbreak.

The key intervention for ending this AdV outbreak was advancing the AdV vaccine to the earliest possible date for newly arriving recruits, in addition to ensuring any recruit remaining on station (including those anticipating separation) were vaccinated. Average time from vaccination date to symptom onset date among AdV cases in training was 0.9 days (SD 9.4 days). Of note, early symptoms of AdV may be very mild, and some individuals were likely already symptomatic with unrecognized AdV when vaccinated, and most cases became symptomatic before the 14 days required to have reached full vaccine effectiveness. Inpatients appeared more likely to have received vaccine while symptomatic, but the clinical significance of receiving vaccine after infection with AdV is beyond the scope of this report. While there was initial question about decreased vaccine effectiveness with this particular AdV strain, the rapid decrease in attack rates, shown in **Table 2** , and outbreak resolution upon implementation of the accelerated vaccination schedule, strongly suggest that the circulating strains of AdV remained covered by the current AdV vaccine (Adenovirus Type 4 and Type7 Vaccine, Live, Oral).

A strength of the study included the availability of multiplex respiratory pathogen PCR for rapid diagnosis of cases. Tracking of cases using Microsoft Excel, MHS GENESIS, and Microsoft Teams, for efficient and secure collection of data and collaboration between MCRD San Diego, NMCSD Preventive Medicine, Navy Environmental and Preventive Medicine Unit FIVE, and the Epi Data Center from the Navy Marine Corps Force Health Protection Command allowed for accurate and efficient expert consultation. Other strengths included the ability to identify vaccination timing and the results of accelerating the AdV vaccine schedule.

Limitations of this study include the delay in case identification, likely underestimation of case numbers, and data limitations on calculating vaccine effectiveness based on person-time. The outbreak was characterized by mild symptoms at illness onset, leading to delays in care seeking and laboratory testing, particularly at the beginning of the outbreak when laboratory testing was potentially not conducted, unless warranted due to pneumonia concerns. The delay in case identification introduced challenges for monitoring outbreak progression and measuring intervention effectiveness. To mitigate this, we analyzed data using both symptom onset and clinic visit dates, using symptom onset date for the epidemiological curve; however, our data could not be used to calculate vaccine effectiveness based on person-time. Despite these gaps in case capture and person-time analysis, vaccination was clearly crucial in controlling the outbreak, preventing severe disease casualties, and preserving the training schedule.

This outbreak demonstrated, despite availability and widespread use of effective vaccines during recruit training, that AdV remains a significant medical threat to military recruits when the vaccine is not administered expeditiously, upon arrival to a recruit training center. Early vaccination should remain a central tenet for prevention and control of communicable diseases in these high risk, congregate settings.
